# Design, Characterization, and In Vivo Application of Multi-Conductive Layer Organic Electrocorticography Probes

**DOI:** 10.1021/acsami.3c00553

**Published:** 2023-05-04

**Authors:** Rémy Cornuéjols, Amélie Albon, Suyash Joshi, James Alexander Taylor, Martin Baca, Sofia Drakopoulou, Tania Rinaldi Barkat, Christophe Bernard, Shahab Rezaei-Mazinani

**Affiliations:** † Mines Saint-Etienne, Centre CMP, Departement BEL, F-13541 Gardanne, France; ‡ Aix Marseille University, INSERM, INS, Inst Neurosci Syst, 13005 Marseille, France; § Department of Biomedicine, Basel University, 4056 Basel, Switzerland

**Keywords:** neural interface devices, capacitive couplings, crosstalks, bioelectronics, neural recordings, thin-film polymers, microelectrode arrays

## Abstract

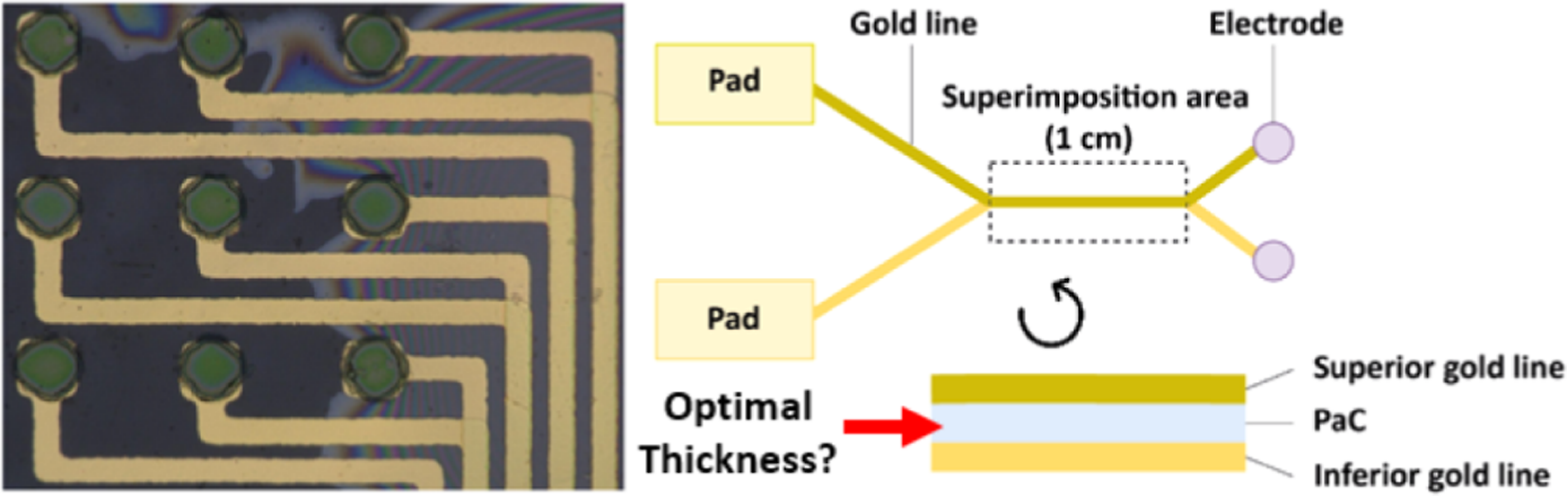

Biocompatible and plastic neural interface devices allow for minimally invasive recording of brain activity. Increasing electrode density in such devices is essential for high-resolution neural recordings. Superimposing conductive leads in devices can help multiply the number of recording sites while keeping probes width small and suitable for implantation. However, because of leads’ vertical proximity, this can create capacitive coupling (CC) between overlapping channels, which leads to crosstalk. Here, we present a thorough investigation of CC phenomenon in multi-gold layer thin-film multi-electrode arrays with a parylene C (PaC) insulation layer between superimposed leads. We also propose a guideline on the design, fabrication, and characterization of such type of neural interface devices for high spatial resolution recording. Our results demonstrate that the capacitance created through CC between superimposed tracks decreases non-linearly and then linearly with the increase of insulation thickness. We identify an optimal PaC insulation thickness that leads to a drastic reduction of CC between superimposed gold channels while not significantly increasing the overall device thickness. Finally, we show that double gold layer electrocorticography probes with the optimal insulation thickness exhibit similar performances *in vivo* when compared to single-layer devices. This confirms that these probes are adequate for high-quality neural recordings.

## Introduction

Monitoring biological events in the brain requires recording of neural activities, which can be achieved with neural interface devices that can be implanted either inside brain regions or on the cortical surface. Such recordings have allowed for significant breakthroughs in the understanding of brain biology, including network oscillations, place cells, and grid cells.^1−4^ The rise of flexible and biocompatible organic materials has led to a rapid growth in their use for neuroscience applications.^5−12^ Mechanically compliant substrate to soft tissue for implantable multi-electrode array (MEA) enabled the fabrication of devices that can conform to the curvatures of the brain surface, thus limiting tissue damage.^12,13^ Thin-film MEA typically consists of polymeric materials, such as polyimide (PI) or parylene C (PaC), for encapsulation together with Au, Pt, or Ir electrodes.^12−17^ In addition, conducting polymers have emerged as prime candidates for interfacing with biological tissue. Coating electrodes with a soft and biocompatible conducting polymer, such as poly(3,4-ethylenedioxythiophene) doped with poly(styrene sulfonate) (PEDOT:PSS), reduces foreign body response to devices, thus enabling acute and as recently reported chronic recording of neural activity.^12,18^ PEDOT:PSS coating leads to a lower impedance, which in turn increases the signal-to-noise ratio and provides higher quality recordings.^19,20^ The semiconductive properties of PEDOT:PSS originate from its delocalized pi electrons. In this polymer, macromolecular blocks are held together by electrostatic and weak van der Waals interactions, which allow hydrated ions to permeate into the polymer’s structure, and thus, an efficient ionic to electronic current conversion occurs. This makes PEDOT:PSS a highly conductive material.^5,21^

Large-scale neural recording with high spatial accuracy is necessary to gain mechanistic insights into some brain functions. Extensive research on thin-film polymer MEA has led to an increase in electrode density, channel count, and optimized device geometry.^11,12,22−25^ Higher electrode count however comes with the augmentation of lead density. This accumulation of leads increases device dimensions, which can become an issue for their implantation. Superimposing conductive leads can reduce device width and provide the opportunity to augment the number of recording sites on neural devices.^26^ However, increasing electrode and lead density increases the risk of crosstalk between channels, which has already been reported in high density polymer MEAs.^27^ Crosstalk between leads can be quantified by generating a signal input on one channel and assessing its presence on other channels.^28−30^ The crosstalk between neighboring tracks depends on at least four factors: effects occurring directly between channels through the polymeric encapsulation, effects occurring between the leads and the surrounding medium, electrode site impedance, and grounding conditions.^31^ There is however no information on crosstalk occurring in multi-conductive layer devices with superimposed tracks, for which leads are much closer and shielding one another from the surrounding medium.

In this work, we characterized the crosstalk occurring between superimposed leads in multi-conductive layer thin-film MEAs. We also studied the effect of insulation layer thickness between overlapping leads on crosstalk to determine the optimal thickness for interference reduction while minimizing the total device thickness. Because of device geometry, the capacitive coupling (CC) occurring between superimposed leads was considered as the dominant crosstalk source. We first fabricated and characterized multiple double gold layer MEAs, containing superimposed leads. We utilized PaC as an insulation layer in these devices and its thickness ranged from 210 to 1627 nm across MEAs. Crosstalk occurring between overlapping leads was systematically measured for the range of insulation thicknesses and was described using theoretical analysis. The determined optimal value for crosstalk reduction was confirmed *in vivo* through the implantation of double gold layer electrocorticography (ECoG) probes over the mouse auditory cortex. This study provides a guideline on the design, fabrication, and characterization of multi-conductive layer thin-film MEAs for high spatial resolution recording.

### Multi-Conductive Layer MEA: Design and Characterization

**Figure 1:**
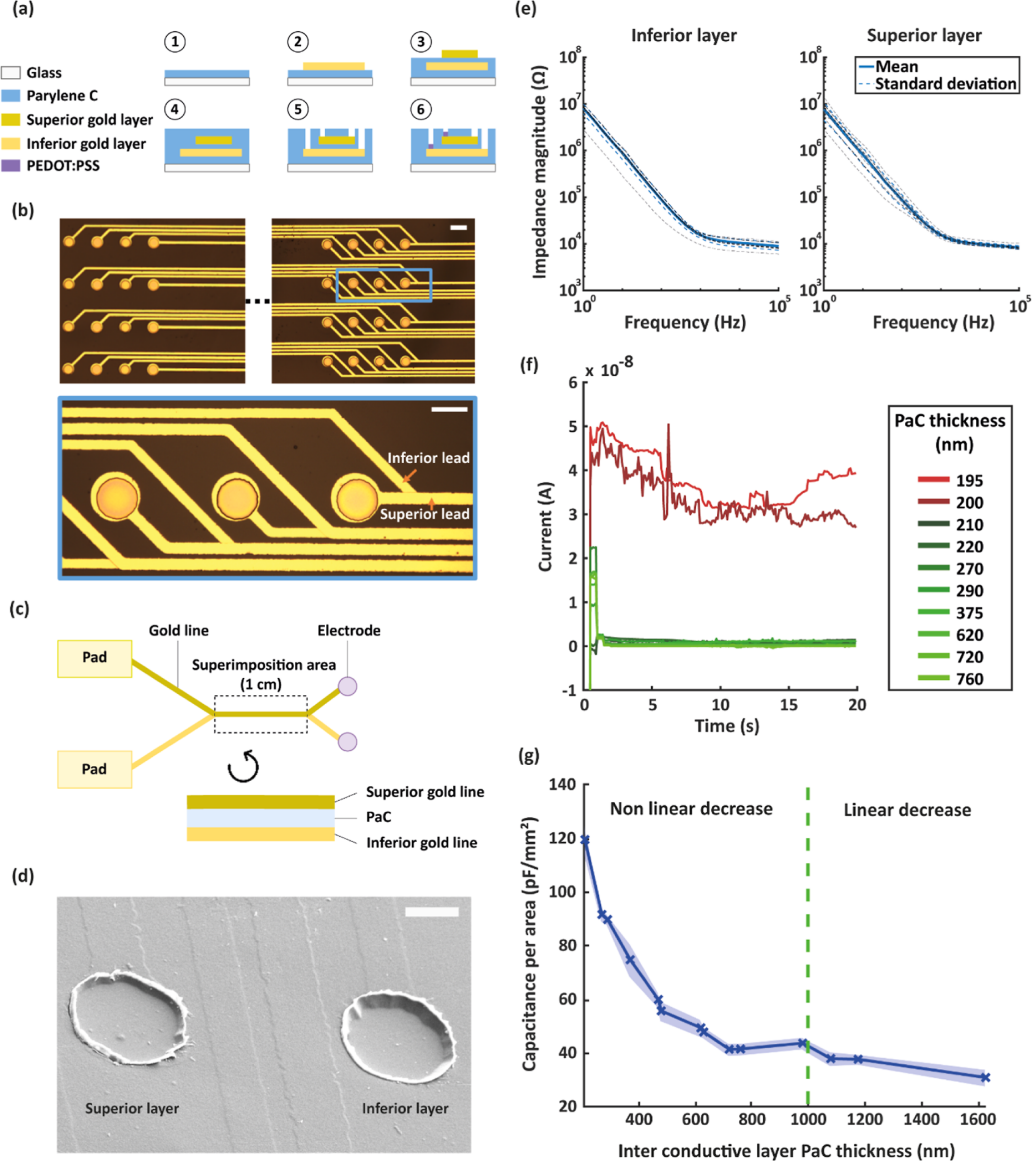
Fabrication process, design, and characterization of a double gold layer MEA. (a) Fabrication procedure (1: PaC deposition, 2: inferior gold layer patterning using photolithography, 3: PaC deposition for insulation and superior gold layer patterning, 4: PaC deposition for encapsulation, 5: reactive ion etching for pad and electrode opening, and 6: PEDOT:PSS patterning using peel-off technique). (b) Pictures of electrodes on the MEA. The device has two groups of electrodes. Zoom on electrodes and superimposed leads are shown in the blue inset. Each electrode from one group has a superimposed lead with the lead of an electrode from the other group. These lines are superimposed over 1 cm and then split to join their respective pads. Scale bars: 100 μm. (c) Schematics presenting the principle of the MEA design, depicted using two electrodes with their respective gold lines and pads. Top view of the full path of gold lines (top part) and cross-sectional view of the 1 cm superimposed area (bottom part). Not to scale. (d) Scanning electron microscopy (SEM) picture of a PEDOT:PSS electrode placed on the superior gold layer (left) and one placed on the inferior gold layer (right), taken with a 45° angle. Insulation layer thickness is 980 nm. The depth difference due to the multi-gold layer design is minor when compared to the size of the electrode. Scale bar: 10 μm. (e) Average impedance ± standard deviation for 20 μm electrodes located either on the inferior (left) or the superior (right) gold layer. The averaged impedance is calculated over 10 electrodes for each depth, and dotted black lines represent individual electrodes’ impedance. Both groups of electrodes have highly comparable impedances. Therefore, depth difference shows no impact on the electrode performance. (f) Pinhole test in different PaC layers, ranging from 195 to 760 nm. The initial current surge is due to capacitive loading caused by the switching of the voltage. PaC layers were pinhole free for films thicker than 210 nm, as can be seen from the low current measured originating from adequate insulation. (g) Normalized capacitance to the superimposition area between two superimposed gold lines, as a function of inter conductive layer insulation thickness. The measurement was carried out using a capacitance meter at 1 kHz. Normalized capacitance has a clear non-linear decay for insulation thicknesses below 800 nm and a linear decay above 1 μm.

We fabricated a multi-conductive layer MEA to characterize CC phenomenon between superimposed leads and to study the influence of insulation layer’s thickness on CC. Two layers of gold with different depths were patterned on these devices. A PaC insulation layer was deposited between those conductive layers to insulate them from one another (Figure 1a). PaC was reported as an excellent insulation material for neural interface devices based on its extremely low leakage current, high insulation impedance, and biocompatibility.^32^ In the device, each electrode had a lead that was either on the first (superior) or the second layer (inferior; Figure 1b). Patterned gold leads on the superior level were superimposed with inferior gold leads over a 1 cm length (Figures 1c and S1). We fabricated 14 MEAs with different insulating PaC thicknesses, ranging from 210 to 1627 nm. The depths of electrodes placed on the two conductive layers varied as much as the thickness of the insulation layer (Figure 1d). This did not affect the performance of electrodes, as it is illustrated by a similar impedance recorded from electrodes placed on either layer for a 980 nm thick insulating layer (Figure 1e). We firstly investigated the presence of pinholes in PaC. Layers of PaC with multiple thicknesses ranging from 195 to 760 nm were deposited on glass slides covered with a 150 nm gold thin film. These samples were immersed in a phosphate-buffered saline (PBS) solution, and a 1 V voltage was applied between the gold and a platinum counter electrode placed in the solution alongside an Ag/AgCl.^33^ The results (Figure 1f) revealed that our PaC deposition procedure (Figure S2) led to pinhole-free insulations for layers thicker than 210 nm. In fact, the presence of pinholes in thinner layers caused higher DC currents due to insulation failure. Additionally, high local current density caused the gold to delaminate on samples with pinholes. The measured current was however stable and in the 10^–9^ A range for layers thicker than 210 nm. We verified the impact of the resistance existing between superimposed leads, which could contribute to leakage current. Resistivity is defined as
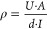
1where *U* is the applied voltage, *A* is the surface immersed in PBS, *d* is the insulation layer thickness, and *I* is the measured current. PaC resistivity was consequently determined in the 10^11^ Ωm range, which implied a resistance between MEA gold leads in the range of hundreds of GΩ. Therefore, resistive effects existing between superimposed leads were negligible. Subsequently, we explored the relationship between normalized capacitance to superimposition area, between the superimposed leads and insulating PaC thickness (measured using a capacitance meter). We discovered that the normalized capacitance decreased as the PaC thickness increased (Figure 1g). Capacitance had a seemingly non-linear decay until 800 nm to 1 μm. Thereafter, there was a slow linear decrease. Consequently, increasing the PaC insulation above 1 μm marginally improved CC, as compared to the CC obtained in the 200–800 nm range. This can be explained by the fact that our device structure, which consists of a double gold layer with PaC insulation (Figure 1c, cross-sectional scheme), created a coupling analogous to a parallel plate capacitor, whose capacitance per area is defined by the following equation
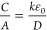
2where *C* is the capacitance, *k* is the dielectric constant of the insulating material, ε_0_ is the vacuum permittivity, *A* is the surface area of the plates, and *D* is the distance between them. PaC is a good insulating material since it has a low dielectric constant, *k* = 3.0 at 1 kHz.^34^ Moreover, concerning the device geometry, the lead dimensions are 18 μm (width) and 1 cm (length), which give a total area of 0.18 mm^2^. We chose such a long length to amplify the CC phenomenon, to be able to measure and characterize it without being hindered by noise. According to eq 2, with these dimensions, the theoretical normalized CC is expected to be 10 pF/mm^2^ at 1 kHz for a PaC thickness of 1 μm. Our measurements of capacitance between superimposed gold lines for multiple insulation thicknesses indeed exhibit values in this range (Figure 1g), in line with the theoretical value. In addition, these results suggest an inversely proportional relationship between the capacitance and insulation thickness indicated in eq 2. Here, we demonstrated that superimposing conductive layers create a CC phenomenon. The increase of PaC thickness decreases capacitance between superimposed leads and has a diminishing impact on CC. According to these measurements, a 1 μm PaC insulation thickness is enough to drastically reduce the CC effect, while not significantly increasing the overall device thickness.

### Assessment of Impact of Capacitive Coupling on Recording

We investigated the effect of CC on electrical recordings, using a customized electronic setup. A pulse generator was used to deliver an input signal on a superior gold lead of the MEAs (Figure 1c). Since we previously identified that there was a capacitance existing between the two conductive layers due to CC, we recorded the crosstalk signal on the inferior gold lead as the setup’s output. We chose sine sweep signal with frequencies ranging from 100 Hz to 10 kHz as an input because action potentials (APs) and local field potentials (LFPs) can have spectral components in these frequencies.^4^ We did not use frequencies below 100 Hz because the signals appeared to be very noisy in this frequency range. Data analysis revealed that the noise existed up to 200 Hz and that it was due to the signal generator. This noise was therefore intrinsic to the stimulation setup used in this experiment and did not depend on the MEA performance. While this setup aimed at characterizing the electrical effect of CC, it is important to note that an *in vivo* device would not have the limitation given by the generator used in this section. The electrical effect of CC was characterized with this experiment, and its consequence on electrophysiological recordings is detailed in the next section.

**Figure 2:**
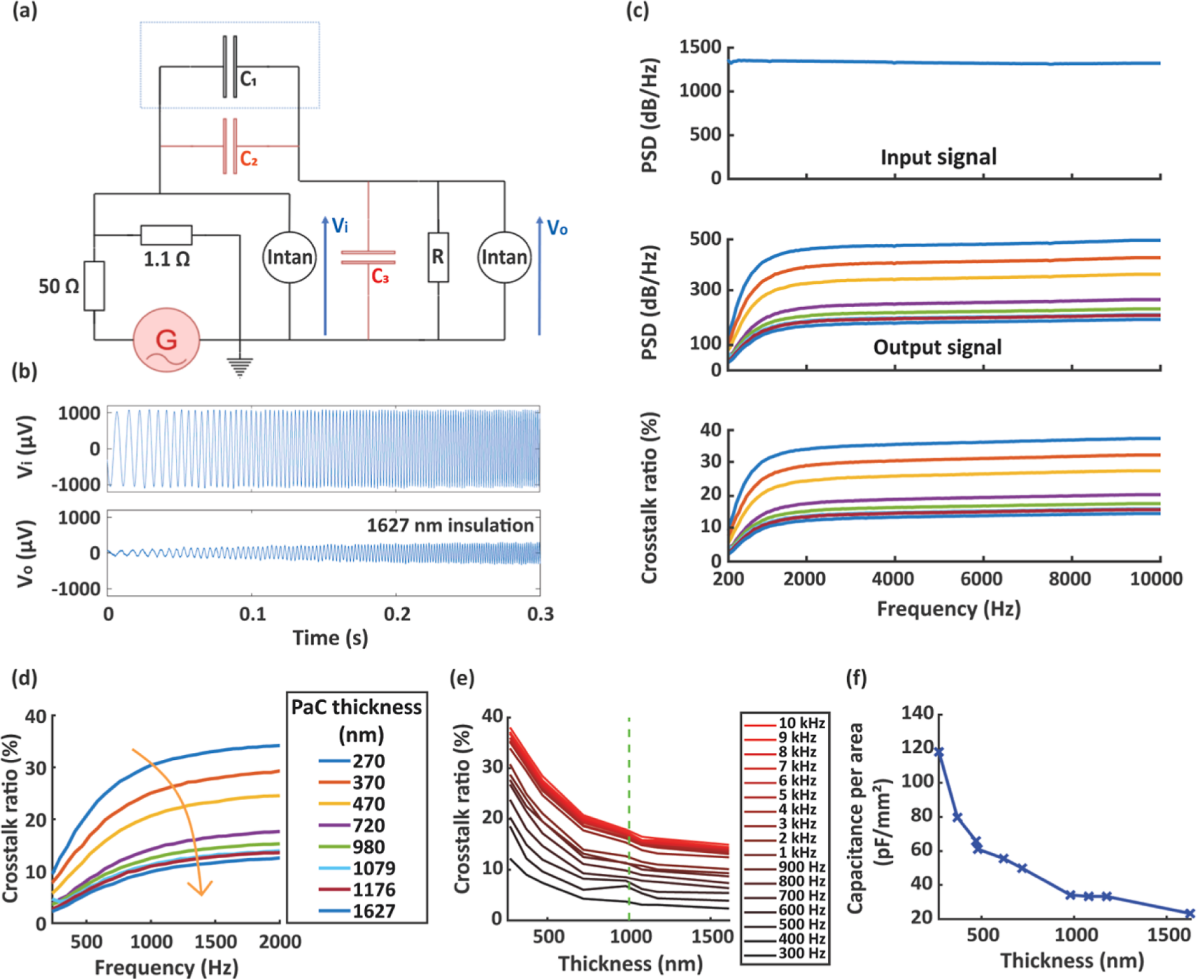
Electronic setup and characterizations for assessing the impact of CC on recordings. (a) Equivalent electronic circuit representation of the setup. The MEA is represented by the dotted line, with the capacitance *C*_3_ describing the CC between superimposed gold lines. *C*_1_ and *C*_2_ represent parasitic CCs occurring between the cables and inside the recording equipment, respectively. *R* is a resistance added for the purpose of measuring the cutoff frequency of a high pass filter effect, created due to the multiple capacitances in the setup. (b) Examples of the first 0.3 s sine sweep signal, applied by the signal generator as the input (top) and of recorded output crosstalk signal (bottom). In this time window, the frequency increases linearly from 100 to 700 Hz. (c) (Top) Frequency domain analysis of the input signal from 200 Hz to 10 kHz, presenting PSD vs frequency. The signal was noisy from 1 to 200 Hz because of the signal generator before becoming stable. (Middle) Frequency domain analysis signal from 200 Hz to 10 kHz of the output signals as a function of PaC thickness—from 210 to 1627 nm. The legend is identical to (d). Responses were noisy between 1 and 200 Hz. The PSD shows an increasing trend before reaching a plateau. (Bottom) Ratio of the two previous PSDs, presenting the same trend as the middle graph. (d) Zoom on the 200 Hz to 2 kHz portion of the ratio of PSDs. The cutoff frequency increases with the inter-gold layer PaC insulation thickness while maximal ratio decreases. (e) Ratio of PSDs as a function of inter-gold layer PaC insulation thickness for multiple frequencies. The ratio decreases with the thickness of the insulation for all frequencies, and increasing insulation thickness decreases less with less effect on CC, until it reaches thicknesses greater than 1 μm. (f) Capacitance per area as a function of inter-gold layer PaC insulation thickness calculated using modeling. These values tend toward 0 and are similar to those measured with the capacitance meter (Figure 1g), which confirms the accuracy of the setup.

The electronic setup consisted of a voltage divider bridge, the MEA, and a resistor. The voltage divider bridge delivered the input signal with a biologically relevant 1 mV amplitude to the input lead (transmitter) on the MEA. The signal was transmitted to its superimposed lead (receiver) through the capacitance *C*_1_ existing due to CC. The receiver lead was then connected in series with a 10 MΩ resistor (Figure 2a). Input (1 mV sine sweep) and output (crosstalk at resistor’s terminal) signals were recorded using an Intan system (Figure 2b). The 500 MΩ internal resistance of this system in parallel with the 10 MΩ resistor creates a 9.8 MΩ equivalent resistor *R*. We predicted that the setup would act as a high pass filter because of the capacitive nature of the coupling. The resistor was introduced to the system for having the cutoff in the range of the frequencies studied here, while not impacting the maximum gain. CCs occurred between wires and within the recording equipment, adding additional capacitances to the system (*C*_2_ and *C*_3_, respectively). *C*_2_ and *C*_3_ acted as extra interference sources in the setup. Recording signals without MEA helped us characterize the significance of these parasitic couplings, with values of *C*_2_ and *C*_3_ at 5.0 and 14 pF, respectively.

After reintegrating the device to the setup, we computed the power spectrum densities (PSDs) of the input and output signals for the MEAs. In order to study the proportion of the input that is transmitted to the receiver lead, due to CC, we calculated the ratio between the input and output PSDs (Figure 2c). Despite the noise existing below 200 Hz, the overall behavior of the system could clearly be visualized. We observed that the maximum PSD was reached at approximately 2 kHz for all devices, as expected from the resistor *R* and the range of the capacitance *C*_1_ values (Figure 1g). The plateau region (after 2 kHz) slowly increased as the dielectric constant grew for higher frequencies, increasing *C*_1_ (eq 2). Additionally, we noted that CC had a high pass filtering effect. Zooming on the region of 200 Hz to 2 kHz (Figure 2d) highlights the most significant features of the filter: with increasing insulation thickness, the cutoff frequency increased while the maximum gain decreased. This also had a diminishing effect on the maximum gain and cutoff frequency, which was expected due to the result of capacitance per area measurements (Figure 1g). We also calculated the PSD ratio as a function of insulation thickness for multiple frequencies, ranging from 300 Hz to 10 kHz (Figure 2e). This analysis revealed that the ratio proportionally decreased with the insulation thickness in keeping with our previous results. A plateau region was reached for 1 μm PaC insulation thickness over all frequencies. These results confirm that 1 μm of PaC is the optimal insulation layer for crosstalk reduction.

To explain the plateau phenomenon of the analyses (Figure 2d,e), we modeled the electronic setup. The high pass filter can be explained using the following transfer function equation
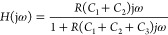
3

This equation implies that the maximum gain of the filter corresponds to
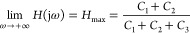
4

Consequently, the gain at cutoff frequency is
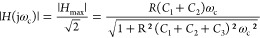
5

Finally, merging (3) and (4), the expression of the cutoff frequency is obtained
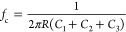
6

According to eq 2, *C*_1_ tends toward 0 when insulation thickness increases. Therefore, with this increase, the maximum gain and cutoff frequency tend toward  and , respectively. Numerically, the limits of the cutoff frequency and the maximum gain are 838 Hz and 0.263, respectively, which implies that the limit of the PSD ratio is 6.9%. These values are consistent with our experimental measurements (Figure 2c). Our findings highlight that the measurement setup has an impact on crosstalk, independently from the MEA. The internal resistance of the recording equipment is inversely proportional to the high pass filter cutoff frequency. Moreover, parasitic CCs occurring in the measurement setup influence both the cutoff frequency and the maximum gain. The amplitude of the crosstalk is highly dependent on these interference sources whether CC occurs between conductive leads in the device or not. Finally, we calculated theoretical *C*_1_ from eqs 5 and 6 for all devices (Figure 2f) based on the cutoff frequency and maximum gain extracted from our measurement results. Then, we compared these values to our initial capacitance measurements (Figure 1g). We observed that the values are approximately similar. The difference between the experimental and theoretical curves is due to the sub-200 Hz noise in the measurements. This comparison suggests that our model was accurate, and it confirms our experimental findings in Figure 2e.

Our results indicate that CCs, originating from the lead superimposition and the measurement setup, create the presented high pass filter effect (eq 3). Efforts should be made to reduce the impact of parasitic couplings to decrease the effect of crosstalk on recordings. On the MEA side, coupling between superimposed leads is directly linked to insulation thickness. An increase in PaC thickness reduces the impact of CC between leads and has a diminishing effect as it becomes thicker. We show that 1 μm of PaC is the optimal insulation layer for crosstalk reduction.

### *In Vivo* Recording Performances of Double Conductive Layer ECoG

**Figure 3:**
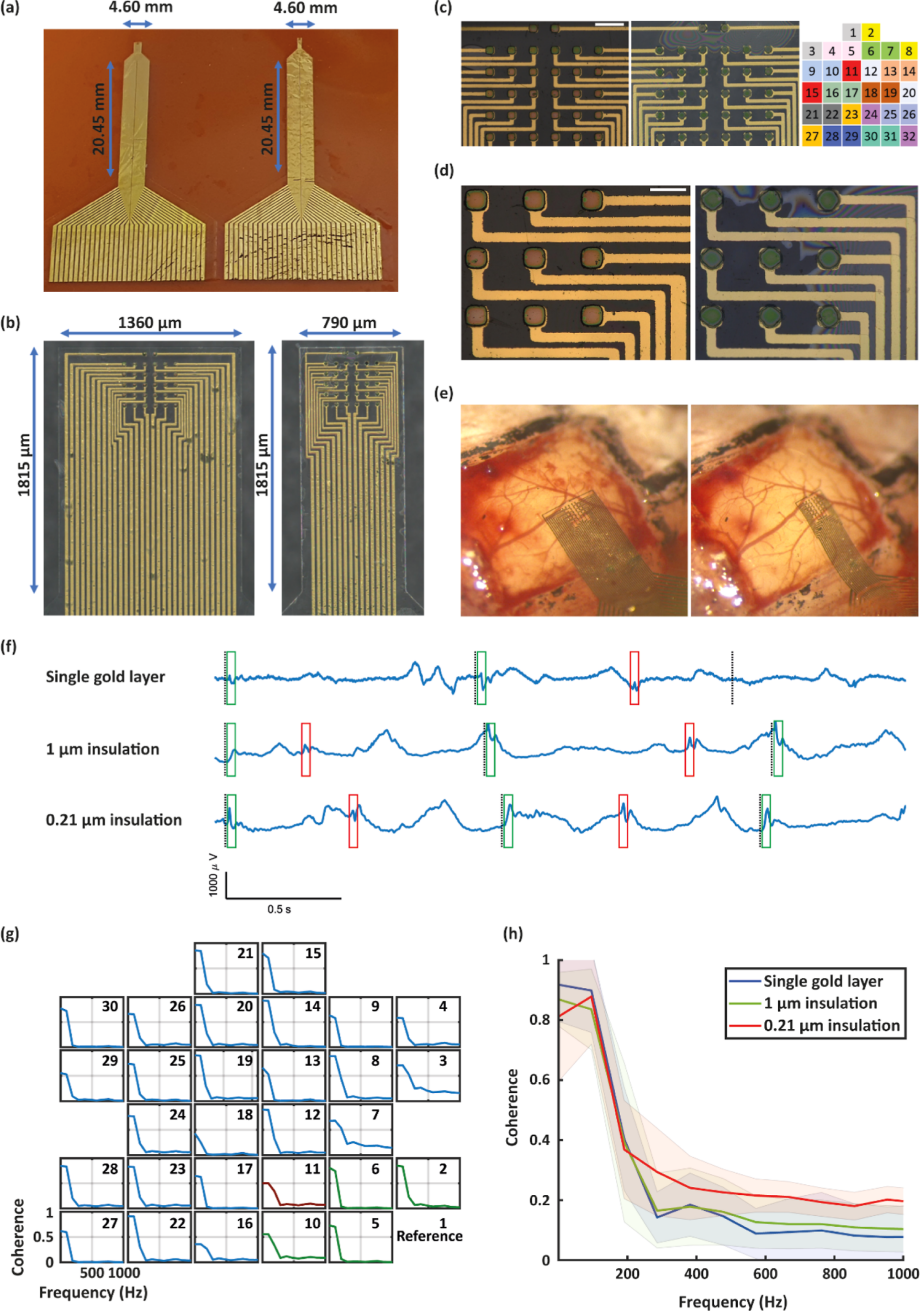
Impact of CC in double gold layer ECoGs during in vivo recording. (a) Picture and dimensions of microfabricated ECoG probes consisting of one gold layer (left) and two superimposed gold layers (right). Overall dimensions are similar, except for the device tip. (b) Zoom on the device tip of the single gold layer ECoG (left) and double gold layer ECoG (right). The double gold layer ECoG has a significantly smaller width in comparison to the single gold layer probe. (c) Zoom on electrode sites located on the tip of single-layer ECoG (left) and double gold layer ECoG (middle). Scale bar: 100 μm. Mapping of the electrodes for data analysis (right). Numbers having the same color represent electrodes having superimposed leads on the double-layer ECoGs. (d) Zoom on electrodes and leads for single (left) and double (right) gold layer probes. Scale bar: 40 μm. The superimposition of leads is displayed on the panel. (e) Pictures of the single gold layer probe (left) and double gold layer probe (right) placed on the dura mater over the mouse’s auditory cortex. (f) 3 s epoch of the electrophysiological signal recorded by an electrode for each implanted ECoG probe: one single gold layer probe, one double gold layer probe with 1 μm insulation between superimposed leads, and one with 0.21 μm insulation. Dotted lines represent onset of auditory stimulations. The green and red rectangles represent evoked and spontaneous activity, respectively. (g) MSC matrix using the bottom right electrode as a reference computed on the 1 μm insulation double gold layer probe. The green curves represent electrodes having leads neighboring the lead of the reference. The red curve represents the electrode having a superimposed gold lead with the reference. Note that electrodes 15 and 20 did not record any activity. Numbering corresponds to the mapping of the electrodes shown (c). If crosstalk occurred between electrodes having neighboring tracks to the reference, their coherence would have higher values than the rest due to signal transfer. This was not observed, and there is therefore no significant crosstalk occurring within this double-layer ECoG. (h) Average raw data MSC between electrodes with superimposed leads on double gold layer ECoG and electrodes having similar positions on the single gold layer ECoG. Averaged over 14 pairs of electrodes. The lack of coherence between the recording sites is similar for single layer and 1 μm insulation double-layer ECoGs, which confirms that 1 μm inter gold layer PaC insulation thickness is enough to prevent CC. With 0.21 μm, high coherence values indicate significant crosstalk. Note that there is a high coherence value for all devices in the 1–200 Hz frequency range. This is due to the volume conduction of low frequencies and cortical activity.

We compared recording performance of single and double gold layer (0.21 and 1 μm insulation thicknesses) ECoG probes *in vivo* (Figure 3a). The lead superimposition reduced the width of devices tip by nearly 50% as compared to single-layer probes (Figures 3b and S3). To record from the same region in the auditory cortex using both designs, we maintained the same spatial distribution of electrodes (Figure 3c). On double conductive layer ECoGs, each electrode had a lead that was either on the superior or the inferior layer. Patterned gold leads on the superior level were superimposed with inferior ones (Figure 3d) over a length, varying from 2 to 3 mm depending on the electrode position. Therefore, the area of superimposition ranged from 0.024 to 0.036 mm^2^. This range of areas allowed us to study its impact on CC (Figure S4). We found that the capacitance created through CC seemingly increased linearly with the area of superimposition, which is compatible with eq 2. Here, the area was minimized by superimposing gold leads only on the implanted part of the probes. We tested two thicknesses for the insulation layer, 0.21 and 1 μm. Probes were placed on the dura mater over the auditory cortex in mice (Figure 3e), and sound stimulation (50 ms stimuli, 20–80 dB) was used to compare the responses measured by the different devices (Figure 3f). Our first objective was to investigate crosstalk occurring within ECoG probes between neighboring tracks. For all devices, the magnitude squared coherence (MSC) between the unfiltered data recorded by each electrode of the matrix and a reference electrode located at the bottom right was calculated from 0 to 1 kHz (Figures 3g and S5). This frequency range was chosen to analyze both the sub-200 Hz signal characteristics of LFPs, as well as their higher frequency components (Figure S6). For all the analyzed MSCs, the peaks observed at sub-200 Hz frequencies can be explained by auditory cortex activity and the volume conduction of low frequencies. We observed similar MSCs for close and far electrodes with respect to the reference (Figure 3g). This similarity is due to the distance between the electrodes placed above the dura and the cortex and the ketamine–xylazine anesthesia leading to the synchronization of spontaneous activity. Moreover, if crosstalk was contaminating recordings because of lead proximity, the MSC between electrodes having neighboring leads to the reference would have higher values because of signal transfer compared to the other electrodes. This was not observed, which implied that there was no significant crosstalk occurring within the double-layer probe between the neighboring tracks. Similar observations were made on the single gold layer ECoG (Figure S5).

Furthermore, to compare the performance of double- and single-layer ECoG probes, we chose pairs of electrodes with the same positions in both designs, which had superimposed leads on the double-layer devices. We studied the presence of CC between overlapping leads by computing MSC between the unfiltered data recorded by these electrodes (Figure 3h). We then averaged these measures for each device over the 14 electrode pairs having recorded electrophysiological data. Averaging helped suppress electrophysiological variabilities between ECoG probes since they might have recorded different groups of neurons even if placed above the same area approximately. In addition, since MSCs were similar for close and far electrodes from the reference in Figure 3g, and since there is no crosstalk occurring between neighboring tracks, the MSC computed between two electrodes did not depend on their distance from one another. MSCs for the studied electrode pairs were consequently equivalent and could be averaged. If CCs were contaminating recordings on double gold layer designs, the average coherence between electrodes having superimposed leads should be higher than the coherence on the single-layer design. This was only observed in the 0.21 μm ECoG device (Figure 3h), for which the average MSC had a significant difference with MSCs from the two other devices above 300 Hz (*p*-value < 0.05). Similar results were found when repeating the experiment (Figure S7). Therefore, our findings confirm that 1 μm PaC insulation layer is the optimal thickness to negate the effect of CC in *in vivo* recordings. These results also show that below this value significant CC starts to affect the measurements.

## Discussion and Conclusions

In this work, we present a method for characterizing crosstalk as well as a guideline for designing thin-film polymer multi-conductive layer devices. We demonstrate that the capacitance created through CC between superimposed leads decreases with insulation thickness, and that it has a linear decay for PaC layers thicker than 1 μm. Moreover, the electronic setup presented here is an efficient tool for characterizing crosstalk. 1 μm was identified as the optimal insulation thickness to reduce crosstalk while not significantly increasing the overall thickness of devices. Finally, this result was confirmed *in vivo*, where double gold layer ECoG with 1 μm PaC insulation thickness did not present any interference between superimposed leads and had a similar performance to a single gold layer ECoG. These probes are therefore adequate devices for neural recordings. Moreover, double gold layer devices had a width that was almost halved as compared to single gold layer devices which entails an easier implantation, especially if this design is applied to intracortical arrays. Stacking leads allows fitting more recording sites on a device while not increasing its width.

Thicker insulation layers could further reduce CC impact but might introduce fabrication challenges, as well as important changes in mechanical properties. Although we used PaC as the insulating material in this study, this method could be applied to any insulating material, such as PI or SU-8, and should yield similar results. In fact, with similar geometry and thicknesses, the parameter that would impact CC is the substrate’s dielectric constant, as observed in eq 2. PaC, PI, and SU-8 have a similar dielectric constant of 3.0, 3.4, and 3.0 at 1 kHz, respectively.^35,36^ The results depicted in Figure 1g can be scaled depending on the material. The threshold thickness between non-linear and linear evolution of the capacitance per area will increase with the dielectric constant. Although we focused on double conductive layer devices, this fabrication method can be expanded to three or more conductive layers. The phenomenon involved should remain similar, with CC occurring between adjacent conductive layers. Moreover, concerning the impact of leads’ superimposition area on CC, efforts should be made to keep it minimal when designing probes. Superimposing leads only on the implanted part of probes, as it was done in this study, can help achieve minimal crosstalk between channels.

When comparing our results to previous studies regarding crosstalk in highly dense neural recording devices, it is important to notice the difference in involved phenomenon. Although we characterized the crosstalk occurring within the devices between superimposed conductive layers, other studies concentrated on neighboring tracks in single conductive layer devices.^27,31^ This implies that not only characterization of effects occurring between leads, such as CC and fringing effect, should be considered but also impedance created between leads and their surrounding environment is critical. In this study, CC was regarded as the only crosstalk source between superimposed leads. In fact, the fringing effect impact was considered negligible compared to that of CC because of leads’ width and proximity, respectively, in the 15 and 1 μm range. This is confirmed by similar results yielded by direct capacitance measurements (Figure 1g) and capacitance computed through electronic setup measurements (Figure 2f). Then, MEAs crosstalk was measured in a dry environment, which entails low interaction between the leads and the devices’ surrounding.^31^ As for ECoG probes, we considered that leads on the inferior layer were shielded from the effect of the surrounding environment by the leads on the superior layer. Additionally, while fringing and impedance from the surroundings might have been present between neighboring tracks in our ECoG probes, they were not significant. This was confirmed by the fact that for the single gold layer ECoG, the coherence between electrodes was similar whether electrodes had neighboring tracks or not (Figure S5). It is also important to note that CC between neighboring leads was considered negligible when compared to the coupling of superimposed tracks. With the distance between neighboring leads and the area of exchange having values in the 10 μm and 100 nm range, respectively, eq 2 predicts that the impact of CC was insignificant when compared to the values of our study. This was validated by our inability to measure a capacitance between neighboring tracks with our measurement tools.

The method presented in this work can be applied to any thin-film polymer multi electrode array with simple changes in fabrication processes. This study provides guidelines on design, fabrication, and characterization of multi-conductive layer bioelectronic devices and implantable probes. This improves the current state-of-the-art organic device fabrication by presenting an efficient method for increasing the number of electrodes with minimal impact on device width. This work paves the way for large-scale neural recordings with high spatial accuracy.

## Methods

### MEA Fabrication

MEAs were designed on AutoCAD 2021 Software. 2 μm of PaC was deposited on a clean 25 mm × 75 mm glass substrate by an SCS Labcoater 2. Detailed information on the PaC deposition procedure can be found in Figure S2. A first metal layer was patterned using a lift-off process with a bi-layer of LOR5A resist and S1813 photoresist. The SUSS MBJ4 contact aligner was used to expose the photoresist. 10 nm of chromium and 150 nm of gold were evaporated with a Boc Edwards thermal evaporator. Lift-off is performed by immersion in dimethyl sulfoxide. Then, a PaC insulation layer was deposited together with the adhesion promotor 3-(trimethoxysilyl)propyl methacrylate (A-174 Silane). A subsequent second layer of metal electrodes and leads was photolithographically patterned and deposited on the glass slide. After lift-off, a 2 μm PaC encapsulation layer was deposited with the adhesion promotor. Then, the electrodes were coated with PEDOT:PSS. This process can be described as the following: a 2 μm sacrificial layer of PaC was deposited above a previously spin-coated soap layer. AZ10XT was photolithographically patterned and developed in AZ developer. Next, the PaC was etched with an Oxford Plasmalab 80 Plus (reactive ion etcher). A solution containing Heraeus Clevios PH1000, ethylene glycol, dodecyl benzene sulfonic acid, and (3-glycidyloxypropyl) trimethoxysilane was spin-coated four times (3000 rpm once and 1500 rpm three times) with a one-minute bake in-between at 110 °C. The sacrificial PaC layer was peeled-off and baked at 140 °C for 1 h. Finally, the device was washed in deionized water to remove any excess of low-molecular-weight compounds. Two array areas, of roughly 0.2 × 2 mm^2^ and equidistant to the center of the design, account for 32 electrodes, each distributed in an 8 by 4 matrix of electrodes (Figure S1). Half of the electrodes have a diameter of 50 μm, while the other half have 20 μm. Leads near the electrode sites were 15 μm wide with 15 μm clearance.

### ECoG Fabrication

The fabrication method of ECoGs was similar to the process described for MEAs with the exception that glass wafers were used as substrates and that these substrates were immersed in water after completion of fabrication in order to release the devices from the wafer. Released devices were kept on KAPTON 500 HN until packaging and implantation. Once fabricated, devices were linked to a custom-made printed circuit board (PCB) using 3M Electrically Conductive Adhesive Transfer Tape 9703. Beforehand, an NPD-36-DD-GS Omnetics connector was soldered to the PCB for connection to recording equipment.

### Device Characterization

Thin-film thickness measurements were conducted by stylus profilometry (Bruker Dektak) for thicker devices. Direct measurements of capacitance were conducted using a PeakTech 2170 Multi-function LCR-Meter. Electrochemical impedance spectroscopy was conducted on a Metrohm Autolab potentiostat/galvanostat instrument.

### Scanning Electron Microscopy

Using Carl Zeiss Ultra55, the secondary electron detector was used to investigate electrode structures. All images were taken at 5 kV voltage.

### Electrical Recordings

Sine sweep pulses were generated using a 3390 50 MHz arbitrary waveform generator (Keithley), and data were recorded using an RHS2116 16-channel Stim/record headstage (Intan technologies).

### *In Vivo* Experiments

All experimental procedures were carried out in accordance to Basel University animal care and use guidelines, approved by the Veterinary Office of the Canton Basel-Stadt, Switzerland. The experiments were performed on three adult (8–9 weeks) male C57BL/6j mice (Janvier, France) under anesthesia. Mice were first anesthetized using ketamine (80 mg/kg) and xylazine (16 mg/kg) by an intraperitoneal injection. Next, the subcutaneous injection of bupivacaine/lidocaine (0.01 mg/animal and 0.04 mg/animal, respectively) was used for analgesia. Finally, the anesthesia was supplemented with ketamine (45 mg/kg), as required through the experimental session. All mice were euthanized using a pentobarbital injection at the end of the experiment. During surgery, mice were head-fixed using a custom-made metal headplate attached to the skull using Loctite glue. Their body temperature was maintained at 37 °C with a heating pad (FHC, ME, USA). The skin over the right auditory cortex was then removed, and the skull was exposed. Next, a craniotomy (approximately 2 × 2 mm^2^) was performed with a scalpel above the right auditory cortex. After removing the bone, the dura was covered with silicone oil for protection. Then, the packaged ECoG electrodes were placed over the auditory cortex above the dura (as shown in Figure 3e) using a motorized stereotaxic micromanipulator (DMA-1511, Narishige, Japan). Sound stimulation and recordings were performed using TDT System 3 (Tucker Davis Technologies, FL, USA). For sound stimulation, stimuli were generated with a digital signal processor (RZ6, Tucker Davis Technologies, FL, USA) at 200 kHz sampling rate and played through a calibrated MF1 speaker (Tucker Davis Technologies, FL, USA) positioned at 10 cm from the mouse left ear. Stimuli were calibrated with a wide-band ultrasonic acoustic sensor (model 378C01, PCB Piezotronics, NY, USA). For recording, the ECoG electrodes were connected using a 32-channel omnetics connector to the PZ5 amplifier (Tucker Davis Technologies, FL, USA). Electrophysiological signals were recorded at a 24414 Hz/channel and digitized using a RZ2 processor (Tucker Davis Technologies, FL, USA). ECoG responses were recorded to continuous white noise stimuli (50 ms, 20 to 80 dB).

### Statistical Analysis

The MSC values of the 14 pairs of electrodes having superimposed leads were analyzed using Mann–Whitney *U*-test. The first test was applied between the 0.21 μm insulation ECoG and the single gold layer ECoG. The second test was applied between the 0.21 and the 1 μm insulation ECoG. The tests were performed for each frequency point above 300 Hz.

### Data Analysis

All analysis was performed using Matlab (Mathworks).

## Data Availability

All data needed to evaluate the conclusions in the paper are present in the paper. Additional data related to this paper may be requested from corresponding authors upon reasonable request.

## Supporting Information Available

Design of the MEA in Autocad; PaC deposition procedure; SEM picture of 1 μm insulation double gold layer ECoG; capacitance created through CC as a function of the area of superimposition between leads; MSC matrix using the bottom right electrode as a reference computed on the single gold layer probe; spectral components of electrophysiological data; and average raw data MSC between electrodes with superimposed leads on double gold layer ECoG and electrodes having similar positions on the single gold layer ECoG (PDF)

## References

[ref1] O’Keefe J. Place Units in the Hippocampus of the Freely Moving Rat. Exp. Neurol. 1976, 51, 78–109. 10.1016/0014-4886(76)90055-8. 1261644

[ref2] Steriade M. Grouping of Brain Rhythms in Corticothalamic Systems. Neuroscience 2006, 137, 1087–1106. 10.1016/j.neuroscience.2005.10.029. 16343791

[ref3] Moser E. I.; Kropff E.; Moser M. B. Place Cells, Grid Cells, and the Brain’s Spatial Representation System. Annu. Rev. Neurosci. 2008, 31, 69–89. 10.1146/annurev.neuro.31.061307.090723. 18284371

[ref4] Buzsáki G. Rhythms of the Brain. online edn; Oxford University press, 2006. 10.1093/acprof:oso/9780195301069.001.0001

[ref5] Rivnay J.; Owens R. M.; Malliaras G. G. The Rise of Organic Bioelectronics. Chem. Mater. 2014, 26, 679–685. 10.1021/cm4022003.

[ref6] Someya T.; Bao Z.; Malliaras G. G. The Rise of Plastic Bioelectronics. Nature 2016, 540, 379–385. 10.1038/nature21004. 27974769

[ref7] Khodagholy D.; Gelinas J. N.; Buzsáki G. Learning-Enhanced Coupling Between Ripple Oscillations in Association Cortices and Hippocampus. Science 2017, 358, 369–372. 10.1126/science.aan6203. 29051381 PMC5872145

[ref8] Shi J.; Fang Y. Flexible and Implantable Microelectrodes for Chronically Stable Neural Interfaces. Adv. Mater. 2019, 31, 1804895 10.1002/adma.201804895. 30300442

[ref9] Taylor I. M.; Robbins E. M.; Catt K. A.; Cody P. A.; Happe C. L.; Cui X. T. Enhanced Dopamine Detection Sensitivity by PEDOT/Graphene Oxide Coating on in Vivo Carbon Fiber Electrodes. Biosens. Bioelectron. 2017, 89, 400–410. 10.1016/j.bios.2016.05.084. 27268013 PMC5107160

[ref10] Boehler C.; Aqrawe Z.; Asplund M. Applications of PEDOT in Bioelectronic Medicine. Bioelectron. Med. 2019, 2, 89–99. 10.2217/bem-2019-0014.

[ref11] Kaiju T.; Doi K.; Yokota M.; Watanabe K.; Inoue M.; Ando H.; Takahashi K.; Yoshida F.; Hirata M.; Suzuki T. High Spatiotemporal Resolution ECoG Recording of Somatosensory Evoked Potentials with Flexible Micro-Electrode Arrays. Front. Neural Circuits 2017, 11, 20 10.3389/fncir.2017.00020. 28442997 PMC5386975

[ref12] Khodagholy D.; Gelinas J. N.; Thesen T.; Doyle W.; Devinsky O.; Malliaras G. G.; Buzsáki G. NeuroGrid: Recording Action Potentials from the Surface of the Brain. Nat. Neurosci. 2015, 18, 310–315. 10.1038/nn.3905. 25531570 PMC4308485

[ref13] Khodagholy D.; Doublet T.; Gurfinkel M.; Quilichini P.; Ismailova E.; Leleux P.; Herve T.; Sanaur S.; Bernard C.; Malliaras G. G. Highly Conformable Conducting Polymer Electrodes for in Vivo Recordings. Adv. Mater. 2011, 23, H268–H272. 10.1002/adma.201102378. 21826747

[ref14] Stieglitz T.; Beutel H.; Schuettler M.; Meyer J.-U. Micromachined, Polyimide-Based Devices for Flexible Neural Interfaces. Biomed. Microdevices 2000, 2, 283–294. 10.1023/A:1009955222114.

[ref15] Pimenta S.; Rodrigues J. A.; Machado F.; Ribeiro J. F.; Maciel M. J.; Bondarchuk O.; Monteiro P.; Gaspar J.; Correia J. H.; Jacinto L. Double-Layer Flexible Neural Probe With Closely Spaced Electrodes for High-Density in Vivo Brain Recordings. Front. Neurosci. 2021, 15, 15 10.3389/fnins.2021.663174. PMC8239195 34211364

[ref16] Lecomte A.; Degache A.; Descamps E.; Dahan L.; Bergaud C. In vitro and in vivo biostability assessment of chronically-implanted Parylene C neural sensors. Sens. Actuators, B 2017, 251, 1001–1008. 10.1016/j.snb.2017.05.057.

[ref17] Kuo J. T. W.; Kim B. J.; Hara S. A.; Lee C. D.; Gutierrez C. A.; Hoang T. Q.; Meng E. Novel Flexible Parylene Neural Probe with 3D Sheath Structure for Enhancing Tissue Integration. Lab Chip 2013, 13, 554–561. 10.1039/C2LC40935F. 23160191

[ref18] Donahue M. J.; Sanchez-Sanchez A.; Inal S.; Qu J.; Owens R. M.; Mecerreyes D.; Malliaras G. G.; Martin D. C. Tailoring PEDOT Properties for Applications in Bioelectronics. Mater. Sci. Eng., R 2020, 140, 100546 10.1016/j.mser.2020.100546.

[ref19] Ludwig K. A.; Uram J. D.; Yang J.; Martin D. C.; Kipke D. R. Chronic Neural Recordings Using Silicon Microelectrode Arrays Electrochemically Deposited with a Poly(3,4-Ethylenedioxythiophene) (PEDOT) Film. J. Neural. Eng. 2006, 3, 59–70. 10.1088/1741-2560/3/1/007. 16510943

[ref20] Rivnay J.; Inal S.; Collins B. A.; Sessolo M.; Stavrinidou E.; Strakosas X.; Tassone C.; Delongchamp D. M.; Malliaras G. G. Structural Control of Mixed Ionic and Electronic Transport in Conducting Polymers. Nat. Commun. 2016, 7, 11287 10.1038/ncomms11287. 27090156 PMC4838877

[ref21] Volkov A. V.; Wijeratne K.; Mitraka E.; Ail U.; Zhao D.; Tybrandt K.; Andreasen J. W.; Berggren M.; Crispin X.; Zozoulenko I. V. Understanding the Capacitance of PEDOT:PSS. Adv. Funct. Mater. 2017, 27, 1700329 10.1002/adfm.201700329.

[ref22] Wang J.; Zhao Q.; Wang Y.; Zeng Q.; Wu T.; Du X. Self-Unfolding Flexible Microelectrode Arrays Based on Shape Memory Polymers. Adv. Mater. Technol. 2019, 4, 1900566 10.1002/admt.201900566.

[ref23] Lee M.; Shim H. J.; Choi C.; Kim D.-H. Soft High-Resolution Neural Interfacing Probes: Materials and Design Approaches. Nano Lett. 2019, 19, 2741–2749. 10.1021/acs.nanolett.8b04895. 31002760

[ref24] Schendel A. A.; Nonte M. W.; Vokoun C.; Richner T. J.; Brodnick S. K.; Atry F.; Frye S.; Bostrom P.; Pashaie R.; Thongpang S.; Eliceiri K. W.; Williams J. C. The Effect of Micro-ECoG Substrate Footprint on the Meningeal Tissue Response. J. Neural. Eng. 2014, 11, 046011 10.1088/1741-2560/11/4/046011. 24941335 PMC4539281

[ref25] Rodger D. C.; Fong A. J.; Li W.; Ameri H.; Ahuja A. K.; Gutierrez C.; Lavrov I.; Zhong H.; Menon P. R.; Meng E.; Burdick J. W.; Roy R. R.; Edgerton V. R.; Weiland J. D.; Humayun M. S.; Tai Y.-C. Flexible Parylene-Based Multielectrode Array Technology for High-Density Neural Stimulation and Recording. Sens. Actuators, B 2008, 132, 449–460. 10.1016/j.snb.2007.10.069.

[ref26] Airaghi Leccardi M. J. I.; Vagni P.; Ghezzi D. Multilayer 3D Electrodes for Neural Implants. J. Neural. Eng. 2019, 16, 026013 10.1088/1741-2552/aae191. 30215607

[ref27] Porto Cruz M. F.; Vomero M.; Zucchini E.; Delfino E.; Asplund M.; Stieglit T.; Fadiga L. Can Crosstalk Compromise the Recording of High-Frequency Neural Signals? In 2019 9th International IEEE/EMBS Conference on Neural Engineering (NER), 2019; Vol. 9( (12), ), pp 924–927. 10.1109/NER.2019.871700

[ref28] Sayed Herbawi A.; Christ O.; Kiessner L.; Mottaghi S.; Hofmann U. G.; Paul O.; Ruther P. CMOS Neural Probe With 1600 Close-Packed Recording Sites and 32 Analog Output Channels. J. Microelectromech. Syst. 2018, 27, 1023–1034. 10.1109/JMEMS.2018.2872619.

[ref29] Rios G.; Lubenov E. V.; Chi D.; Roukes M. L.; Siapas A. G. Nanofabricated Neural Probes for Dense 3-D Recordings of Brain Activity. Nano Lett. 2016, 16, 6857–6862. 10.1021/acs.nanolett.6b02673. 27766885 PMC5108031

[ref30] Lopez C. M.; Andrei A.; Mitra S.; Welkenhuysen M.; Eberle W.; Bartic C.; Puers R.; Yazicioglu R. F.; Gielen G. G. E. An Implantable 455-Active-Electrode 52-Channel CMOS Neural Probe. IEEE J. Solid-State Circuits 2014, 49, 248–261. 10.1109/JSSC.2013.2284347.

[ref31] Qiang Y.; Gu W.; Liu Z.; Liang S.; Ryu J. H.; Seo K. J.; Liu W.; Fang H. Crosstalk in Polymer Microelectrode Arrays. Nano Res. 2021, 14, 3240–3247. 10.1007/s12274-021-3442-8. 34394850 PMC8361849

[ref32] Xie X. Z.; Rieth L.; Tathireddy P.; Solzbacher F. Long-term in-vivo Investigation of Parylene-C as Encapsulation Material for Neural Interfaces. Procedia Eng. 2011, 25, 483–486. 10.1016/j.proeng.2011.12.120.

[ref33] Hassler C.; von Metzen R.; Stieglitz T. Deposition Parameters Determining Insulation Resistance and Crystallinity of Parylene C in Neural Implant Encapsulation. In 4th European Conference of the International Federation for Medical and Biological Engineering; Vander Sloten J., Verdonck P., Nyssen M.; Haueisen J., Eds.; Springer Berlin Heidelberg: Berlin, Heidelberg, 2009; pp 2439–2442.

[ref34] Kahouli A. Étude des Propriétés Physico-Chimiques et (di)- Électriques du Parylène C en Couche Mince. Ph.D. Dissertation, Université de Grenoble, France, 2011. https://theses.hal.science/tel-00627040.

[ref35] Dupont. Dupont Kapton Polyimide Film General Specifications, Bulletin GS-96-7. http://www.dupont.com/kapton/general/H-38479-4.pdf (accessed June, 2022).

[ref36] Kayaku Advanced Materials. SU-8 for Dielectrics in Organic TFT Back Planes, https://kayakuam.com/products/display-dielectric-layers/ (accessed June, 2022).

