# Supplementary material for: Design, Characterization, and In Vivo Application of Multi-Conductive Layer Organic Electrocorticography Probes
Source: ACS Appl Mater Interfaces. 2023 May 4;15(19):22854–63. doi: 10.1021/acsami.3c00553 (PMC10197075; doi:10.1021/acsami.3c00553)
Supplement: Supplementary file 1 — am3c00553_si_001.pdf [file am3c00553_si_001.pdf]

Supporting Information

Design, characterization and *in vivo* application of multi-conductive layer organic electrocorticography probes

Rémy Cornuéjols[1,2†], *Amélie Albon*[1†], *Suyash Joshi*[3], *James Alexander Taylor*[3], *Martin Baca*[1], *Sofia Drakopoulou*[1], *Tania Rinaldi Barkat*[3], *Christophe Bernard*[2*], *Shahab Rezaei-Mazinani*[1*]

[1] Mines Saint-Etienne, Centre CMP, Departement BEL, F-13541 Gardanne, France

[2] Aix Marseille University, INSERM, INS, Inst Neurosci Syst, 13005 Marseille, France

[3] Department of Biomedicine, Basel University, 4056 Basel, Switzerland

[†]Authors contributed equally to this work

[*]Correspondence: christophe.bernard@univ-amu.fr, charles.rezaei@emse.fr

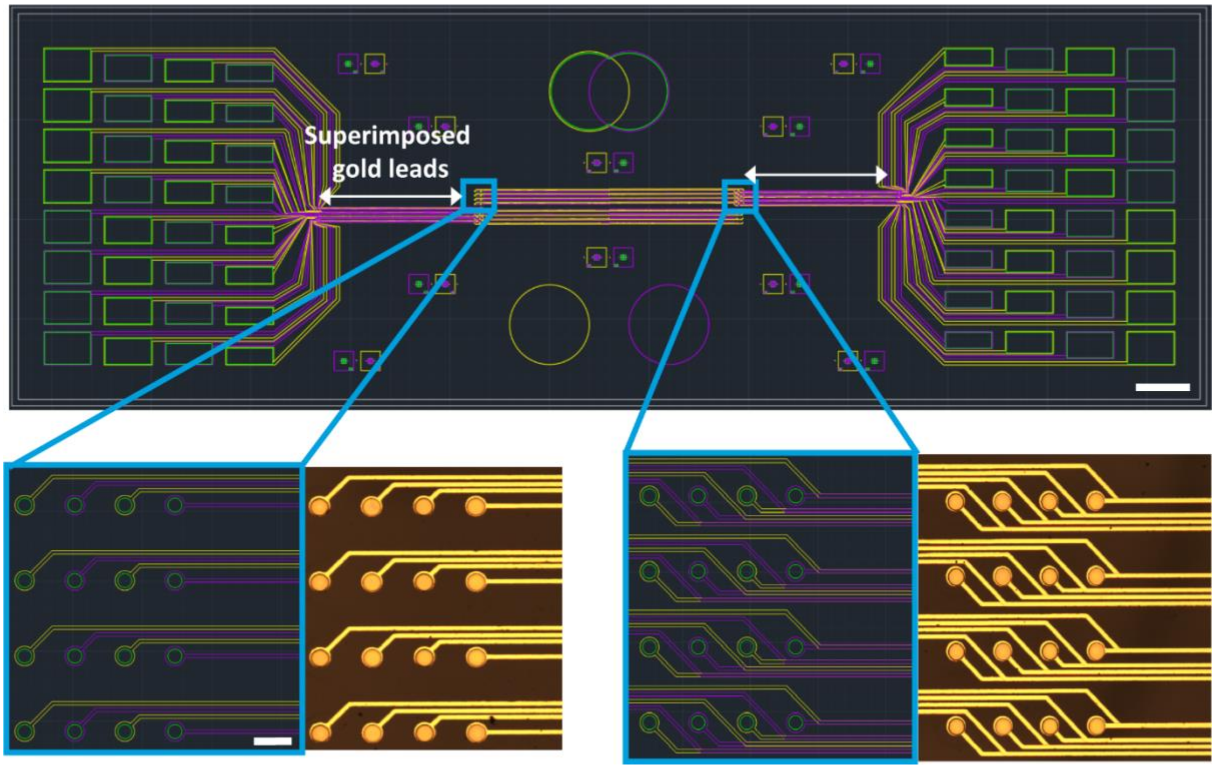

Figure S1: Design of the MEA in Autocad. Yellow and purple lines represent gold patterns on different layers. Gold leads are superimposed over a 1 cm length. Scale bars: 3 mm, 130 µm inset.

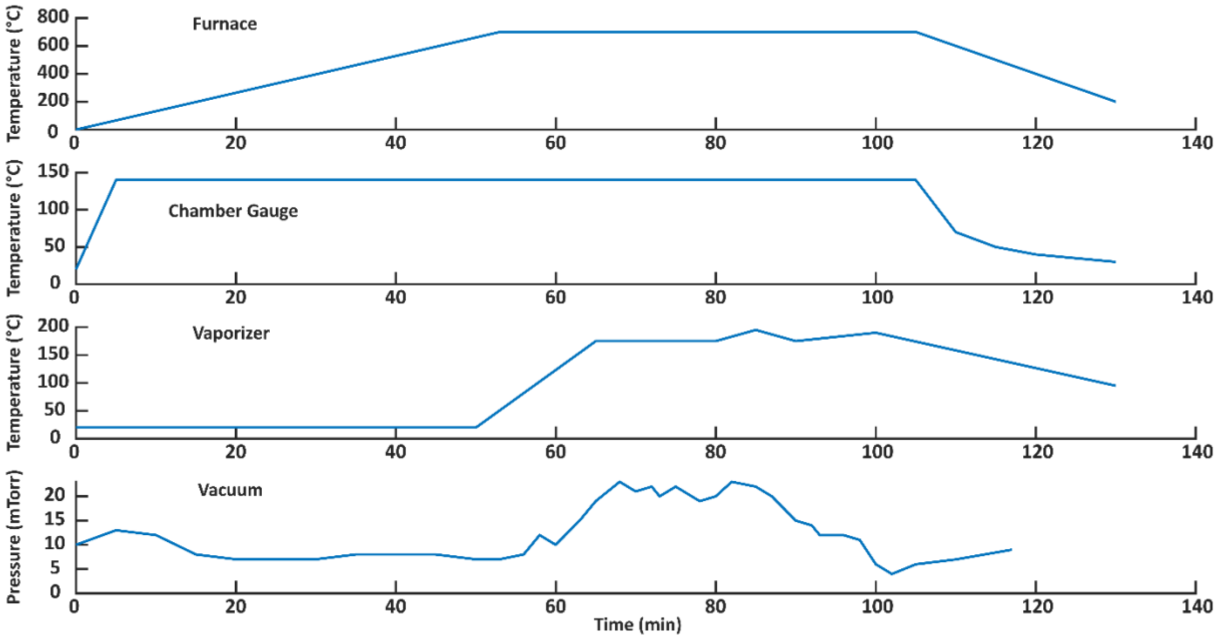

Figure S2: Parylene C deposition procedure. This procedure leads to a pinhole-free PaC deposition for layers thicker than 210 nm. Once the chamber is under vacuum, the furnace heats up to 700°C over 50 minutes. When it reaches its maximum temperature, the vaporizer starts heating. When the vaporizer, containing PaC dimers, reaches 170°, PaC becomes a dimeric gas, which is then pyrolized and cleaved into monomers in the furnace. The monomers then fill the

chamber and deposit on substrates as a transparent polymer film. Pressure in the machine stays under 22 mTorr during the process.

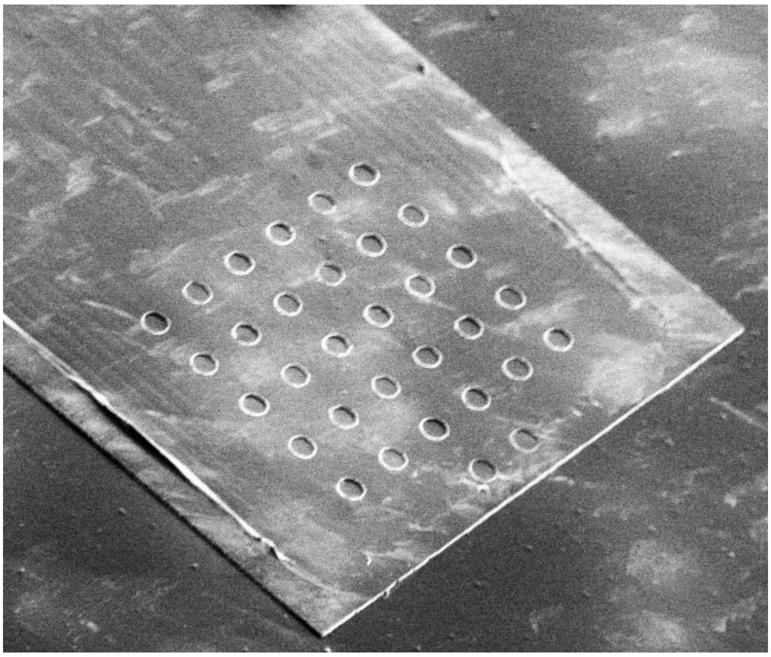

Figure S3. Scanning Electron Microscopy picture of 1 μm insulation double gold layer ECoGs taken with a 45° angle.

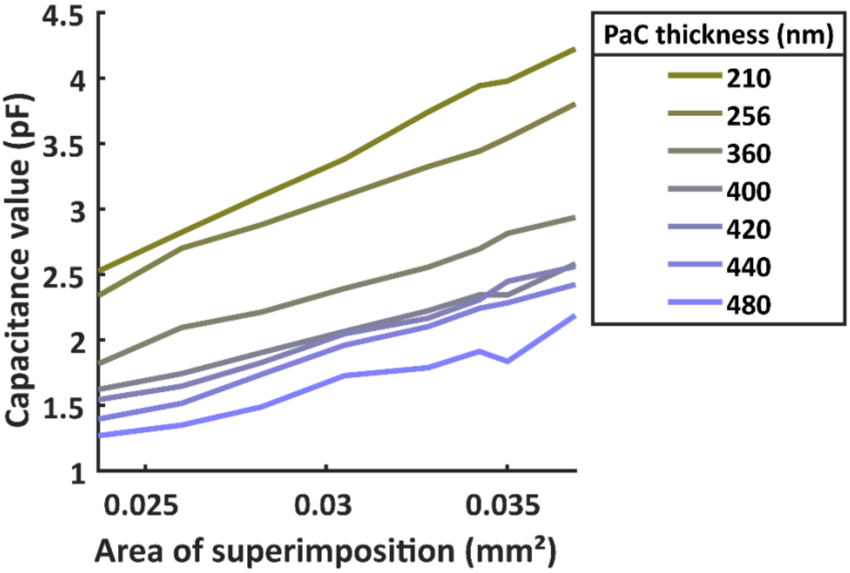

Figure S4: Capacitance created through CC as a function of the area of superimposition between leads. Measured with a capacitance meter. Measurements were averaged over two pairs of superimposed leads for double gold layer ECoGs with PaC insulation thickness ranging from 210 to 480 nm. There is a linear dependency of the capacitance with regards to area of superimposition.

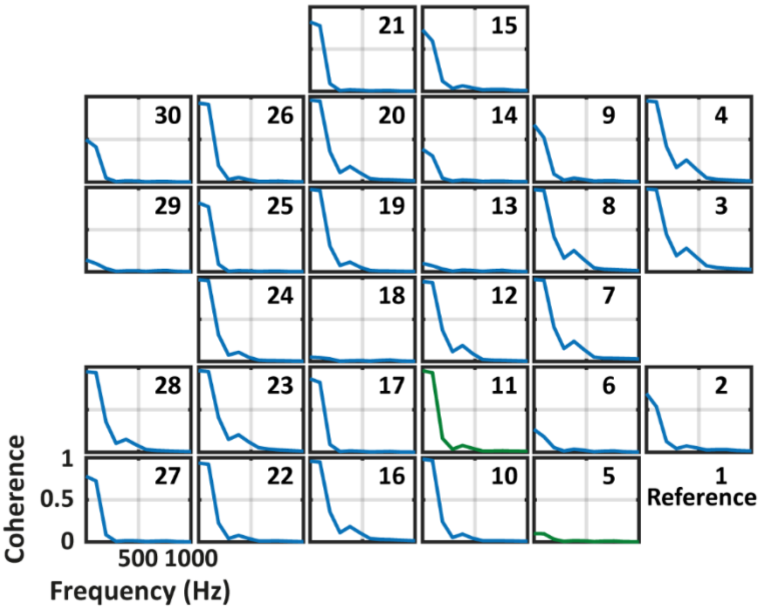

Figure S5: Magnitude squared coherence matrix using the bottom right electrode as a reference computed on the single gold layer probe. Numbering corresponds to the mapping of the electrodes shown Figure 3-c. The green curves represent electrodes having leads neighboring the lead of the reference. Note that electrodes 15 and 20 did not record any signal. If crosstalk occurred between electrodes having neighboring tracks to the reference, their coherence would have higher values than the rest due to signal transfer. This was not observed, there is therefore no significant crosstalk occurring within the single layer ECoG.

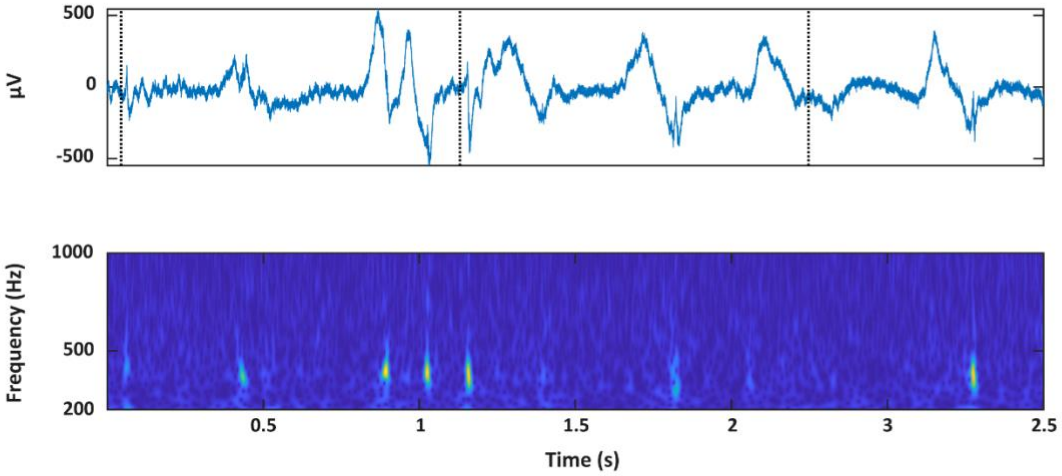

Figure S6: Spectral components of electrophysiological data. Top: 3 seconds epoch of electrophysiological signal recorded by an electrode on the single gold layer ECoG. Dotted lines represent onset of auditory stimulations. Bottom: Gabor Wavelet Transform between 200 Hz and 1000 Hz of the signal above. The signal has spectral components up to 700 Hz.

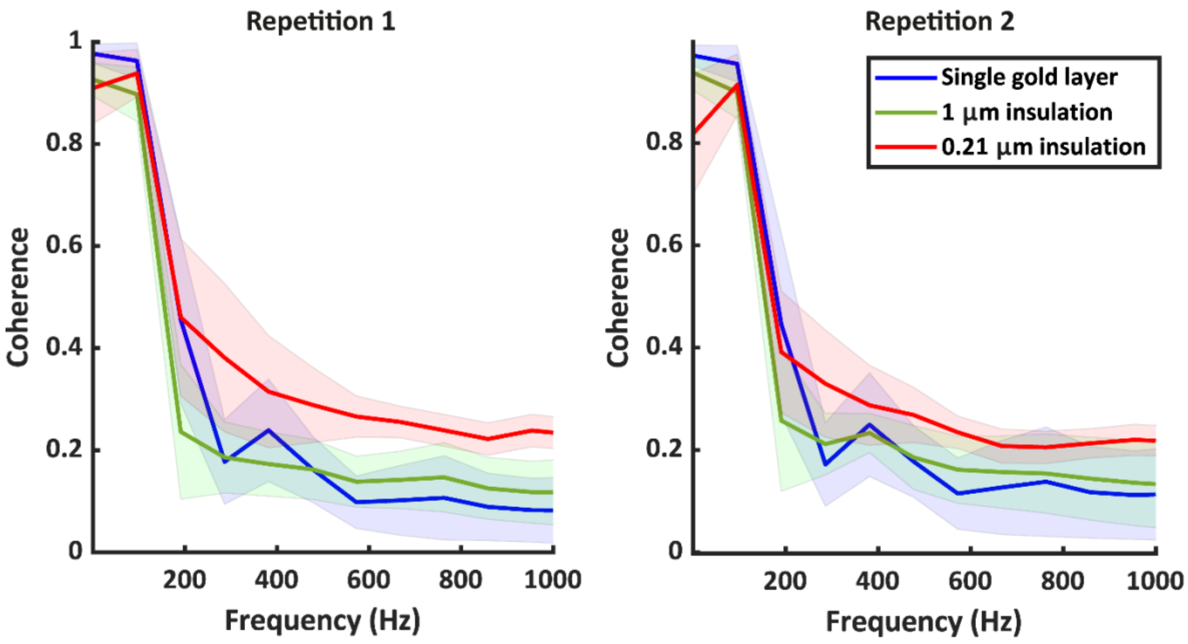

Figure S7: Average raw data magnitude squared coherence between electrodes with superimposed leads on double gold layer ECoG and electrodes having similar positions on the single gold layer ECoG. Averaged over 14 pairs of electrodes, repeated over two experiments. In the same manner as the results shown Figure 3-h, the lack of coherence between the recording sites is similar for single layer and 1 µm insulation double layer ECoGs, which confirms that 1 µm inter gold layer PaC insulation thickness is enough to prevent capacitive coupling. With 0.21 µm, high coherence values indicate significant crosstalk (p-value < 0.05 above 300 Hz).
